# Exploration of the Dynamic Evolution of Online Public Opinion towards Waste Classification in Shanghai

**DOI:** 10.3390/ijerph20021471

**Published:** 2023-01-13

**Authors:** Yingxia Xue, Honglei Liu

**Affiliations:** 1 Management Science and Engineering, School of Economics and Management, Tongji University, 4800 Caoan Rd., Shanghai 201804, China; 2 Department of Construction Management, Changshu Institute of Technology, Changshu 215500, China

**Keywords:** Shanghai, waste classification, dynamic evolution, sentiment analysis, topic modeling

## Abstract

Shanghai is one of the fastest-growing metropolises and the first city in China to implement mandatory waste classification. Waste classification policy of Shanghai has attracted widespread attention since its implementation in July 2019. However, previous papers have not focused on online public attitudes surrounding the implementation of a waste classification policy in Shanghai. In order to fill this gap, this paper explored the dynamic evolution of online public attitudes towards waste classification in Shanghai by using sentiment analysis technology and topic modeling technology. It was found that the proportion of negative posts each month was about 20%; therefore, online public sentiment towards waste classification in Shanghai was generally positive. Compared with the first three months of policy implementation, the public sentiment towards Shanghai’s waste classification became more positive, with the exception of two special periods. Negative posts in July 2019 mainly discussed waste’s environmental hazards and policy provisions. New topics in negative posts in later months focused on some specific problems, including the process of throwing away wet waste, the allocated throwing times, the number of waste cans, takeaway meal disposal, and gathering activities. Improving the factors causing the negative sentiments in the posts will help the government better implement the policy. The paper will help the government to receive higher public support for the waste classification policy in Shanghai. The present findings also have great reference significance for other cities.

## 1. Introduction

According to the 2020 “National Annual Report on the Prevention and Control of Environmental Pollution by Solid Waste in Large and Medium-sized Cities”, issued by the Ministry of Ecology and Environment of the People’s Republic of China, 196 large and medium-sized cities generated 235,602 million tons of domestic waste in 2019. Some developed cities are almost entirely surrounded by waste. Shanghai is a fast-growing metropolis with a population estimated at nearly 25 million [1]. The domestic waste generated per person, per day in Shanghai is close to 1.3 kgs, and the total amount of domestic waste generated per day is close to 32,000 tons, which has put huge pressure on the government. Inadequate waste disposal not only does great harm to the environment [2,3], but also wastes numerous resources [2,3,4]. Mixed waste collection makes it difficult to recycle the waste collected and brings about various problems [5]. Because waste classification and recycling are environmentally friendly and economically efficient [6,7], waste classification is an effective solution to the problem of waste disposal [8].

“Waste classification” was first proposed in an article entitled “The Urban Area Will Separately Collect Garbage”, published in the *Beijing Daily* on 12 December 1957 [9]. As of 2016, the effects were not ideal. On 15 June 2016, the National Development and Reform Commission and the Ministry of Housing and Urban-Rural Development jointly issued a document clarifying that, by the end of 2020, the coverage rate of domestic waste classification collection should reach more than 90% in the key cities, where compulsory waste classification is implemented. In July 2017, the Ministry of Finance, the Ministry of Housing and Urban-Rural Development, the Ministry of Agriculture, and the Ministry of Environmental Protection jointly issued a “Notice on the Comprehensive Implementation of PPP Model in Sewage and Waste Treatment Projects Participated by the Government” [10], to regulate the market operation of sewage and the waste treatment industry, with the aim of improving market efficiency and attracting social capital [11]. On 31 January 2019, the second session of the 15th Shanghai Municipal People’s Congress passed the “Regulations of Shanghai Municipality on the Management of Domestic Waste”, which came into effect on 1 July 2019. Violators would be punished in Shanghai. The compulsory waste classification policy in Shanghai was regarded as the “most stringent” waste classification action in history, and thus caused heated discussion. The domestic waste in Shanghai is classified into recyclable waste, hazardous waste, wet waste, and dry waste [12,13]. Recyclable waste mainly includes paper, plastics, glass products, metals, fabric, and other domestic waste that is suitable for recycling. Hazardous waste refers to waste that causes direct or potential harm to human health or the natural environment, such as batteries, medicines, paint, etc. Wet waste refers to perishable biomass waste, such as food waste, leftovers, expired food, green plants, etc. Dry waste refers to the other waste which does not belong to recyclable waste, hazardous waste, or wet waste.

Text has been used to record information, express opinions, or communicate with others for a long time [14]. As more and more text data become available, text mining technology is widely used to extract unknown or potentially useful information, trends, or rules by processing unstructured data sources [15,16]. Text mining technologies have been efficiently applied in many fields, such as financial [17,18], educational [15], medical [19,20], and environmental domains [10,21], and so on. The previous studies related to waste classification mainly applied questionnaire surveys to investigate residents’ willingness surrounding, and perception of, waste classification, before its implementation. In contrast, text mining technology can be used to collect and process a large amount of text data to obtain public opinions. The data and findings of text mining are more objective and reliable [10,22,23]. Different cities in China have different policies and implementation statuses of waste classification. It is necessary to learn from other cities. Shanghai is the first city in China to implement a mandatory waste classification policy. Previous research has not focused on the dynamic evolution of public attitudes towards Shanghai’s waste classification policy after its implementation. The present study supplements the previous research from this perspective.

The rest of the paper is organized as follows. Section 2 presents a literature review on public participation in waste classification. Section 3 describes the methodology used in the study. Section 4 mainly concerns online data collection and processing. Section 5 presents analyses of public sentiment and dynamic evolution. Section 6 presents the topic modeling analysis for negative posts and compares the topics of different periods. The results are further discussed in Section 7. Section 8 presents the conclusions of the paper.

## 2. Literature Review on Public Participation in Waste Classification

Waste classification can reduce the problems caused by waste disposal and enhance the efficiency of waste recycling [24]. Some scholars have conducted research on public participation in waste classification. Zhuang et al. put forward in 2008 that the Chinese public’s enthusiasm about participating in waste classification was at a low level [25]. Cheng and Urpelainen [26] conducted a survey of 450 respondents to analyze public opinion on waste management in Dar es Salaam. They found that satisfaction with waste management services is related to preference of government leadership. Zhang and Lai [27] explored the relation between intention and household waste classification behavior, using a questionnaire survey considering the psychological behavioral antecedents and circumstantial constraints among Chinese residents. Nainggolan and Pedersen [28] conducted quantitative research to assess households’ preferences for different waste classification and handling schemes, based on a nation-wide survey across Denmark. Minelgaitė and Liobikienė [29] conducted comparative research on waste sorters and non-sorters in separate EU countries, finding that knowledge about general and personal waste generation influenced classification behavior either negatively or insignificantly. Fan and Yang [30] built a “motivation–intention–behavior” model on household solid waste classification and found that environmental motivations substantially affected behavioral intention. Arantes and Zou [31] evaluated the impact of Aifen, an environmental non-governmental organization (NGO), in the context of municipal solid waste management and found that collaborative governance could improve public participation in waste classification. Cudjoe and Yuan [32] evaluated how residents’ awareness of the benefits and difficulties of waste classification affected their intentions, and analyzed the role of policy in influencing residents’ intentions and decisions, as related to waste classification. Liu and Osewe [33] studied the factors influencing residents’ waste classification behavior and environmental protection awareness among the rural residents of Jiangsu, China. Wang and Hao [34] found that perceived environmental governance was important for individual waste classification behavior. Ling and Xu [35] analyzed how social context affected public participation in incentive programs of household waste classification, based on theories of social influence. Wu and Zhang [10] collected text data from the Sina Weibo platform and applied text mining technology to analyze public opinion on the waste classification policy. They put forward that the proportion of people with negative emotions reached nearly half. Liu et al. [36] collected data from 378 ordinary residents and built a mechanism model to study the influence of public education on residents’ willingness to classify household waste. Zhang and Hu [37] applied planned behavior theory and attitude–behavior–condition theory to explore the critical factors affecting residents’ waste classification intention and behavior, finding that attitude had the largest direct effect on domestic waste classification intention.

The previous studies on public participation in waste classification mainly focused on behavior and willingness. These studies mainly adopted a questionnaire survey methodology. It can be found that the sample sizes in questionnaire surveys were generally from dozens to hundreds, and thus the sizes of samples were limited. Questionnaire survey needs a long time and high economic cost to collect data [38,39]. Moreover, questionnaire surveys cannot analyze the public’s real-time sentiments towards policy [40]. Posts in social media usually express posters’ real-time sentiments. The previous papers did not focus on the dynamic evolution of public opinion on Shanghai’s waste classification. This paper collected more than 40 thousand valid posts (after cleaning) on the Sina Weibo platform. Text mining was then performed to analyze the dynamic evolution of public attitude towards Shanghai’s waste classification. The sample size of the study was much larger, so the findings obtained here are more objective.

## 3. Methodology

**Figure 1:**
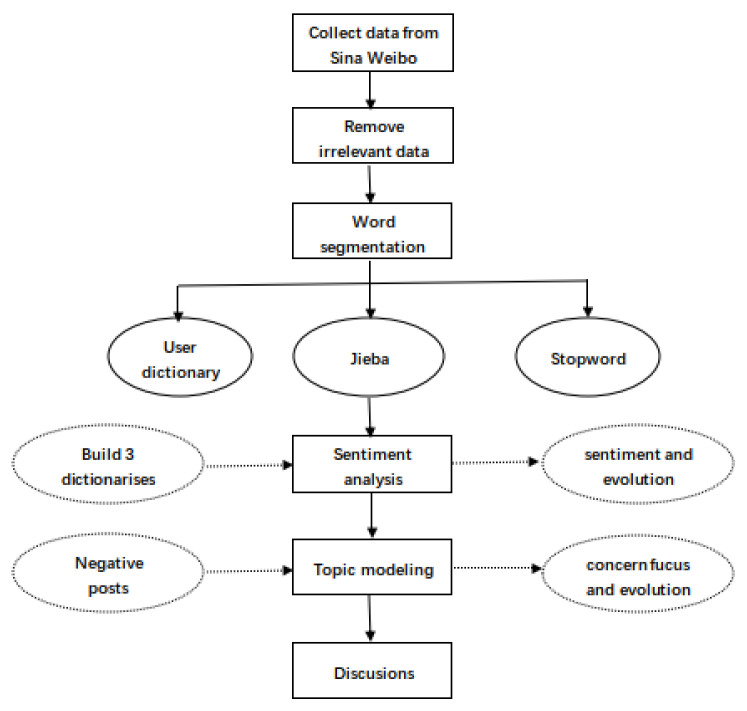
Flow chart of text data processing.

Text mining technology is defined as the computer technology that processes a large amount of text data to discover important or valuable information [41]. Text mining technology is efficient in analyzing the text information systematically [42,43]. Social media platforms make it possible for people in different areas to communicate online, regardless of long distances between them. Social media platforms have been used by many residents to maintain relationships, disseminate information, advertise business, and share opinions [44]. Social media platforms have become an indispensable tool in people’s lives. In recent years, social media are serving as critical data source to collect public opinion [14]. When some residents want to express their own thoughts on certain topics (such as experiences, social hot spots, etc.), they can publish posts on a social media platform to share their views and exchange ideas with others. Posts can be quickly read and spread online by many residents. Social events which are relevant to people’s lives often attract many participants to the related discussions on social media platforms. Therefore, numerous text data have been posted on social media platforms expressing residents’ opinions, which makes it possible to explore public opinion regarding a range of topics, using text mining technology [45,46,47]. Sentiment analysis is a powerful technique for identifying the sentiment of text data. Sentiment analysis can judge the sentiment of a post. Based on the sentiment, posts could be classified into different types. Sentiment analysis has become an active research topic, not only in computer science, but also in social and management science because of its value to society and businesses [48]. The study applied Lexicon-based methods to obtain the sentiment score of each post. It analyzed the text documents one by one. The words in a file were processed word by word. Each word in the text file was compared with words in dictionary files, where sentiment words and their sentiment values were listed [49]. Sentiment analysis was performed to explore the overall sentiment and the dynamic evolution of the public sentiment on waste classification in Shanghai. Topic modeling is another important technique for analyzing text data. Topic modeling is increasingly being used to extract topics from a large, unregulated set of texts [50,51]. Generally speaking, topic models generate several topics, which are composed of a set of keywords, to represent the main meaning. The study applied latent Dirichlet allocation (LDA) to conduct topic modelling for the collected posts. LDA is a typical topic modeling technique [52]. The results of LDA model contain essential statistical information and describe all documents briefly. Thus, the LDA technique can generate the topics in large text data automatically, and is an effective text mining technology [53,54]. The topics of negative posts were analyzed in the present paper to identify reasons for the negative sentiments on Shanghai’s waste classification. This study chose Python 3.8 to code the sentiment analysis and topic modelling. The text data were collected and processed according to flow chart shown in Figure 1. The detailed processing of sentiment analysis and topic modelling analysis is described in Section 5 and Section 6. The implementation of the waste classification policy in Shanghai requires all residents to comply. The results of the policy implementation are affected by residents’ behavior to a large extent. Waste classification is closely related to the daily life of every resident and depends on the behavior of residents [27]. On one hand, the problems encountered by residents with waste classification cause various inconveniences in residents’ lives [55], and thus weakens their willingness to comply [36]. On the other hand, the problems lead to residents engaging in illegal behaviors, which hinders the smooth implementation of the waste classification policy. This paper explores online public opinions on waste classification and the dissatisfactory aspects of residents’ opinions; aiding in the clarification and resolution of the residents’ problems, in the specific operation of waste classification. In this way, residents might more consciously classify waste in accordance with relevant regulations, and thereby, waste classification policies might be better implemented.

## 4. Data Collection and Processing

### 4.1. Data Collection and Cleaning

In order to explore the dynamic evolution of public attitude towards waste classification in Shanghai, from the implementation of waste classification to the present, this study collected the relevant posts on the Sina Weibo platform. “Waste classification in Shanghai” were chosen as the keywords to search posts. In recent years, WeChat Circle, QQ Zone, and Sina Weibo have become the top three social media platforms for Chinese internet users [56]. Among them, Sina Weibo is the only platform where posts are available for all visitors, even if they are not friends. In China, online posts will be processed after being reported by others and confirmed by relevant authorities. Objective and truthful posts can be easily published online in China. Both positive posts and negative posts can often be seen on social media platforms. In this paper, positive posts and negative posts were both analyzed in following sections. When any keywords are searched on the Sina Weibo platform, the relevant posts, including posts of anonymous strangers, will be shown. Visitors who do not log on the platform can only browse the first page of results when searching any keyword. Logged-in users can browse the first 50 pages. However, all posts about waste classification in Shanghai far exceeded 50 pages, so this study adopted the advanced search function of the Sina Weibo Platform, which could be used to search the first 50 pages within a limited time range. In order to collect related posts, this study adopted advanced search functions and adjusted the time range. However, by reading the collected posts, we found that a large part of the posts did not focus on Shanghai’s waste classification. For example, some posts contained comments on waste classification, but were related to some other cities, rather than Shanghai.

The collected text data should be cleaned before further text mining analysis. The noisy objects in the raw data influence the accuracy of results, so it is of great importance to remove them [14,57]. Removing unnecessary content can help to analyze text data more efficiently. In this study, unqualified data were removed, such as duplicate posts, advertisements, and irrelevant posts. After data cleaning, 46,056 valid posts remained, which only accounted for about a third of the total number of all posts. Therefore, cleaning raw text data is very important for obtaining reasonable results. The following analyses are based on the clean text data.

### 4.2. Word Segmentation and Word Frequency

Mining text data in the platform can reveal the public’s opinion [58]. Text mining technology is generally based on statistical analysis of words or phrases. The spaces between English words cause each English sentence to be divided into different parts. However, Chinese sentences are usually composed of several Chinese characters, and there are no spaces between different characters or vocabularies in a sentence. Therefore, when processing Chinese text, we need to segment each sentence into different parts. 

Jieba word segmentation can perform efficient word graph scanning, based on the prefix dictionary [23]. A directed acyclic graph (DAG), which is composed of all possible word-forming conditions of Chinese characters in the sentence, is generated. Dynamic programming is applied to find the maximum probability path and the maximum segmentation combination. For unknown words in the system, an HMM model is used, based on the word-forming ability of Chinese characters.

The study added a user dictionary for word segmentation, mainly including “dry waste”, “wet waste”, “kitchen waste”, “recyclable waste”, the names of some agencies, and other fixed expressions. User dictionaries should be built according to different topics. It can improve the rationality of word segmentation, and thus is of great significance for the subsequent processing.

After word segmentation, it is necessary to get rid of some words, vocabularies, and symbols that often appear but cannot express actual meaning and feelings. These words are listed in a document named “stop words”. In English text mining, stop words usually include “a”, “the”, “in”, “before”, etc. Chinese sentences include similar stop words. The times that each word or phrase appears in all posts can be counted. The highest frequent words and phrases are usually the hottest themes. Reading the highest frequent vocabularies can help to check whether word segmentation is reasonable. If some phrases with high frequency are split into several parts, this indicates that it is necessary to improve the word segmentation results. These expressions should be included in the user dictionary. If some words or symbols that have no actual meaning exist in the text data, it will affect the results of the subsequent sentiment analysis and topic modeling analysis. This type of vocabulary and symbols should be added to the stop word list. Therefore, word frequency statistics analysis is necessary, to check whether word segmentation is reasonable. If the vocabularies appearing in the high-frequency vocabulary list are correct, and there are no wrong vocabularies or meaningless expressions, the word segmentation is reliable. The following analysis can be conducted on the word segmentation results. Table 1 shows the top 60 frequency vocabularies of all posts in the clean text data. Life, work, community, fines, and environment are among the most frequent words. Therefore, the relationships between the five aspects and waste classification are those which the public is mostly concerned about.

## 5. Sentiment Analysis

When processing a large amount of text data, it is hard to judge the overall sentiment of the whole text data set. By conducting sentiment analysis, the sentiment score of each post can be obtained. Sentiment analysis assigned different emotional scores, between −10 and 10, to each word or phrase, which has positive or negative emotions. When the emotion expressed by a word is positive, its score is greater than 0, such as “beautiful”, “like”, etc. When the emotion of a word is negative, its score is less than 0, such as “ugly”, “boring”, etc. At the same time, the influence of negative words and degree adverbs should also be considered. The sentiment score of each post depends on the total score of different words in the post.

According to the sentiment scores, all posts can be classified into different sentimental polarities, such as positive, negative, and neutral [59]. When the total sentiment score of a post is larger than 0, the total sentiment score of the post is positive. When it is lower than 0, the sentiment is negative. When the total sentiment score is 0, the sentiment is regarded as neutral. Based on all sentiment scores of the whole text data, the overall sentiment of the public opinion can be analyzed. It needs to be emphasized that the emotion of the same word or phrase may change under different topics, such as the word “waste”, mentioned in all posts of the study. The word “waste” often has negative implications, but it has no obvious emotional implication here, because of the topic of the paper. The sentiment dictionary compiled here includes about 9600 different vocabularies and scores of each word or phrase. It required a substantial amount of effort to compile the sentiment dictionary for the study. Compiling sentiment dictionaries, according to different topics, is very important to improve the accuracy of sentiment analysis, especially for some vocabularies, which appear very frequently in text data. The dictionaries applied in sentiment analysis of the study included a sentiment word dictionary, a degree word dictionary, and a negative word dictionary.

**Figure 2:**
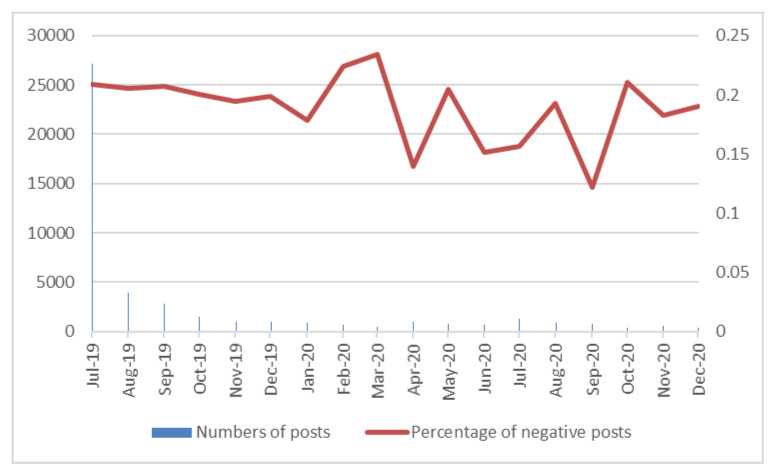
Percentage of negative posts from July 2019 to December 2020.

After conducting sentiment analysis, the sentiment score of each post was obtained. The percentage of negative posts is shown in Figure 2. It can be seen that the proportion of negative posts was about 20% each month, which showed that public attitude towards waste classification in Shanghai was generally positive or supportive. With the exception of three months (February 2020, March 2020, and October 2020), the proportion of monthly negative posts was relatively stable, and presented a slightly downward trend. The proportion of negative posts in the remaining months was lower than the proportions during July–September 2019. Through observing the highest frequency words, it was found that volatilities of the three months—February 2020, March 2020, and October 2020—were caused by some special reasons. Table 2 shows the top 40 most frequently used vocabularies of negative posts from February to March 2020. It could be seen that the frequencies of epidemic-relevant keywords were high during the period, such as epidemic, mask, and isolation. There were 277 negative posts during February–March 2020, and the epidemic-related posts accounted for about one-fifth. February–March 2020 was the most severe stage of COVID-19 (coronavirus disease 2019) prevention and control in China. During the period, many residents expressed their negative sentiments on COVID-19 when they published posts about waste classification. Thus, the emotional fluctuations during this period were mainly affected by the epidemic. The top 20 most frequently used vocabularies of negative posts in October 2020 are listed in Table 3. It can be seen that “Nanjing” was among the top 10 words used. Nanjing started to implement waste classification in October 2020. Although these posts include “Shanghai”, the posts were mainly related to the tension and worries about the implementation of waste classification policy in Nanjing. There were only 77 negative posts in October 2020, of which one-tenth were related to Nanjing’s waste classification policy. When not considering Nanjing-relevant posts, the percentage of the remaining negative posts in October 2020 was lower than the percentage of the first three months. Therefore, the public sentiment towards Shanghai’s waste classification policy was generally positive. Except for the fluctuations caused by the two special events, the percentages of negative posts in other months were lower than the first three months of policy implementation.

## 6. Topic Modelling

LDA is a generative probabilistic model consisting of three layers including vocabulary, topic, and document. It processes a set of posts and generates latent topics [60]. LDA treats a topic as a multinomial distribution of different vocabulary, and a document is considered as a vector of word counts [23]. The distributions of topics and keywords are generated by reversely simulating the document generation process [52]. LDA seeks word clustering by maximizing word co-occurrence probability, and describes the document generation process by Dirichlet distribution.

Topic modeling was applied on the posts with negative sentiments to explore the themes and reasons that affected the negative sentiment. July 2019 was the first month of waste classification implementation in Shanghai, so the residents did not understand Shanghai’s waste classification policy sufficiently. Residents had many doubts about which type different waste belonged to. The number of posts in July 2019 was 27,192, accounting for over half of the total number of posts from July 2019 to December 2020. July 2019 was the most immature period, when various problems appeared. Both the residents and government personnel were at the learning stage and needed to make lots of adjustments. Because topic modeling has a great relationship with word frequency, this study first conducted topic modelling on the negative posts in July 2019. Then, topic modelling was applied on the negative posts of August 2019–December 2020. The study compared the difference between them so as to analyze dynamic evolution of public focus.

**Figure 3:**
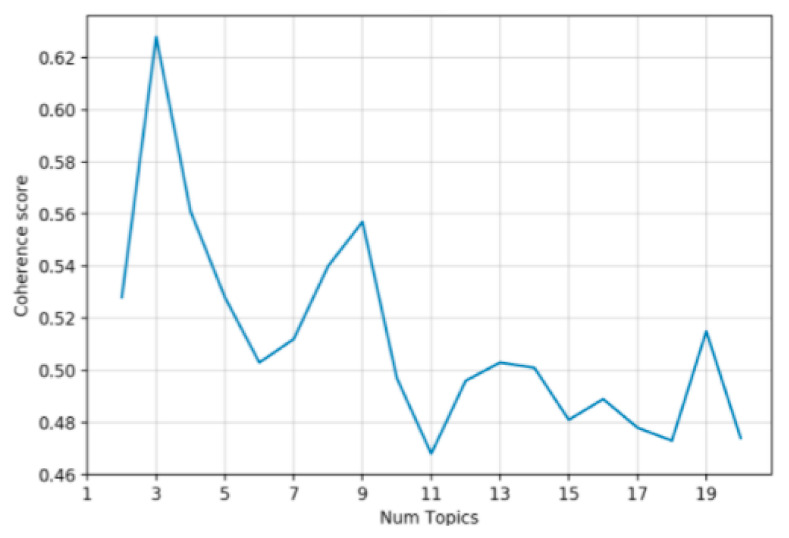
Topic number and coherence score in July 2019.

In this study, coherence score was taken as standard for selecting the optimal number of topics. The relationship between the number of topics and the coherence score of negative posts in July 2019 is shown in Figure 3. It can be seen that, when the number of topics was three, the coherence score was the highest. Therefore, the optimal topic number was set as three. The keywords of three topics were shown in Table 4. Topic 1 was mainly about residents’ confusion about the waste classification policy at the early implementation stage. Most of the residents were not familiar with the new policy during this period, and thus had great doubts about which categories different waste belonged to. Some residents also discussed the relevant problems of waste classification in other cities. The findings of Topic 1 were similar to the findings of Wu and Zhang [10]. Topic 2 was related to the public’s discussion on the necessity of waste classification. Many residents realized that waste classification is very urgent. If the policy was not implemented, the environmental degradation will cause society to pay a higher price. Residents’ environmental awareness affected their behavior surrounding the waste classification procedures [61]. Waste classification has been regarded as a new fashion. Everyone should start from themselves, so as to promote the waste classification implementation in Shanghai. Topic 3 was mainly related to people’s worries about receiving punishment when waste was misclassified. For example, many residents mistakenly believed that the difference between dry waste and wet waste depended on whether the waste was wet or not.

**Figure 4:**
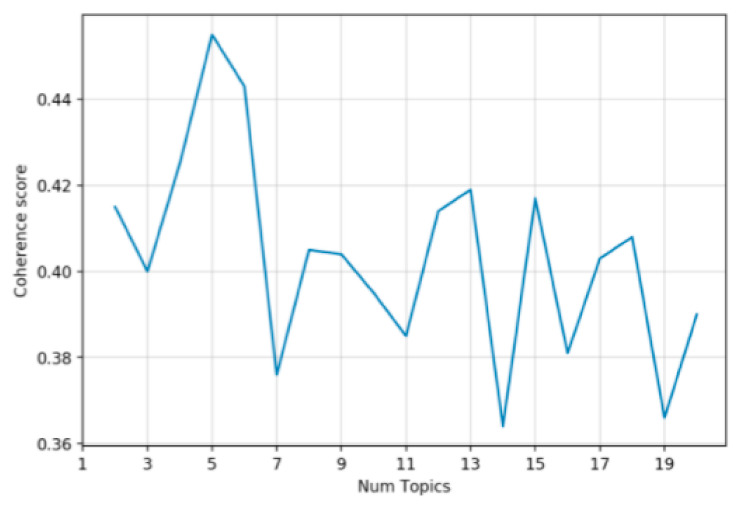
Topic number and coherence score from August 2019 to December 2020.

Figure 4 depicts the relationship between the number of topics and the coherence score of negative posts from August 2019 to December 2020. When the number of topics was five, the coherence score reached the highest, so the optimal number of topics was selected as five. The keywords of each topic are shown in Table 5. It can be seen from the keywords that the first three topics were very similar to the topics of July 2019, while Topic 4 and Topic 5 showed great differences. Topic 4 was mainly about the discussion of wet waste and the waste throwing time [10]. Leftover food usually needs to be packed in plastic bags and brought to the required waste throwing sites. According to Shanghai’s waste classification policy, plastic bags belong to dry waste, and food is wet waste. When throwing wet waste, residents need to open the plastic bags, put the food in the wet waste can, and throw the plastic bags into the dry waste can. It might cause their hands or clothes to be soiled by spoiled food, and thus the inconvenience brings about a negative sentiment in many residents. Residents are required to throw waste in a fixed time frame, which means that a lot of residents who got off work late often miss the waste throwing time, leading to an accumulation of waste in their houses. Moreover, wet waste easily goes bad and emits an unpleasant smell. Therefore, throwing waste away during a fixed time period is a topic which was often complained about in negative posts. Topic 5 was mainly about waste cans, takeaways, and concerts. After the implementation of the waste classification policy, the waste bins are only set in some places, which has brought some inconveniences to people’s lives. There are fewer waste bins in public places, especially wet waste bins. This might be because wet waste can produce unpleasant smells and has a negative impact on the appearance of public places. It becomes less convenient to buy takeaway meals, because the leftovers from the takeaway belong to wet waste. Throwing away wet waste might be more troublesome in some places than others. In addition, the problem of waste classification caused by concerts has also attracted public attention. Many people gather at concerts; therefore, concerts might generate a great deal of waste. Attendees should pay attention to the waste classification of these gathering events.

## 7. Discussion

It can be seen from Table 4 that the posts in July 2019 were mainly discussions on policies and different types of wastes, which was mainly caused by the residents’ insufficient understanding of relevant policies in the first month of the implementation of the policy. As shown in Table 5, the topics were more diversified. The new topics mainly reflected specific problems with waste classification management. In the early implementation period of the waste classification policy, most residents did not understand how to classify different kinds of waste in Shanghai. They lacked the relevant knowledge, and thus were worried about throwing away waste in the wrong way and being fined. In the next few months, new topics appeared in the topic modeling results of negative posts, mainly including wet waste throwing, throwing time, the number of trash cans, takeaway meals, and concerts, etc. Topics 4–5 in Table 5 were new topics. These negative factors were not about the policy itself. The new negative factors were mainly about specific issues, including throwing away wet waste, the throwing time, the number of waste cans, takeaway meals, and large-scale gathering activities, etc. Based on the above aspects, we put forward the following recommendations for relevant departments:

(1) The decision of when the waste throwing time will be needs to consider busy residents. Wet waste might easily go bad, which often produces unpleasant smells and liquids. When throwing wet waste away, residents may stain their clothes and bodies. The need to throw waste away in the allocated time makes busy residents miss the waste throwing time, which causes waste to accumulate in their houses. It is recommended that the waste throwing time is appropriately extended, or that a time period which is convenient for busy residents to throw waste away is added. This could prevent wet waste from going bad in residents’ homes and can reduce the inconvenience of wet waste throwing.
(2) The insufficient number of waste bins makes it difficult to find waste bins to throw waste away in some places. Relevant management personnel should add some waste bins in public places, such as streets and parks.
(3) For concerts and other large gathering events, the organizers should remind attendees of waste classification before the event starts and should sort the waste at the venues after events.

Convenience is an important factor for waste classification, which has been verified by Bernstad [55] and Wu and Zhang [10]. One of the direct reasons for the negative posts was, to a large extent, the inconvenience caused by waste classification. This study found that the inconvenience caused by wrong classification was the issue which was most complained about in the first month, due to insufficient knowledge of classifying waste in Shanghai. After several months, the inconvenience caused by throwing waste away was complained about more than issues of classifying waste. It could be seen that the public’s dissatisfaction with the inconvenience caused by Shanghai’s waste classification mainly focused on throwing wet waste, allocated throwing time, the number of waste cans, and takeaway meals, etc. As shown in Table 4 and Table 5, the potential for being fined was discussed very often by the public and thus is important for waste classification. It was similar to the findings of Chen et al. [62] The factors causing inconvenience were deeply analyzed and discussed in the paper. Wet waste, the number of waste cans, and takeaways were rarely discussed in the previous studies. In addition, it was also found that the waste classification of gathering activities should receive more attention.

## 8. Conclusions

With the acceleration of industrialization and the improvement of living standards in recent years, waste disposal has become an urgent problem in China. Shanghai is the first city in China to implement compulsory waste classification, which has brought about widespread discussion. In recent years, social media platforms are used widely by millions of users to express their opinions on a specific topic [63], which forms a huge amount of text data with valuable information on social media platforms [14,43]. The study aimed to use text mining technology to explore the online public opinion on Shanghai’s waste classification since the implementation of waste classification policy in July 2019. The study firstly collected posts from the Sina Weibo platform and cleaned the collected posts. Sentiment analysis technology and topic modeling technology were then applied, to mine the dynamic evolution of online public attitude towards Shanghai’s waste classification. The main conclusions included the following:

(1) The proportion of negative posts in each month was about 20%, so public attitude towards waste classification in Shanghai was generally positive.
(2) Compared with the first three months after the waste classification policy was implemented, the proportion of positive posts was higher in most of the later months (with the exception of three months when special events occurred). It meant that the public attitude was more positive than the attitude in the first three months. The previous studies related to waste classification did not analyze the dynamic change of public sentiment in long and continuous periods.
(3) The negative sentiment of the posts in July 2019 mainly focused on the negative impact of waste on the environment and waste classification policy.
(4) In the next months, new negative factors appeared in the topics of negative posts, mainly including throwing wet waste away, the allocated throwing time, the number of waste cans, takeaway meals, and large-scale gatherings and activities, etc. The allocated throwing time has been put forward by Wu and Zhang [10]. The other factors have rarely been discussed in the previous studies.

The findings of this study have the potential to help the government understand the public sentiment towards Shanghai’s waste classification in all aspects. Taking corresponding measures would be effective to obtain higher public support for Shanghai’s waste classification in the future. The contribution of this paper is in its exploration of the dynamic evolution of public opinion towards waste classification in Shanghai, by combining big data and text mining technology. After analyzing the public sentiment scores in different time periods, not only the overall attitude of the public, but also the dynamic evolution of public attitudes in consecutive periods was identified. This paper used topic modeling to further analyze the influencing factors of negative attitudes in different periods, and then put forward corresponding suggestions. This study can help the Shanghai government obtain higher public support for waste classification policy. Different cities adopt different waste classification policies, and public opinions vary in a large degree. Therefore, it is important for different cities to learn from each other.

## Figures and Tables

**Table 1:** High frequency vocabularies of all posts.

No.	Vocabulary	Frequency	No.	Vocabulary	Frequency
1	waste	131,798	31	throw wrongly	2964
2	classification	103,113	32	recyclable	2927
3	Shanghai	62,072	33	community	2850
4	life	16,455	34	activity	2847
5	city	9072	35	compulsory	2819
6	waste can	6958	36	new fashion	2778
7	implement	6736	37	implement	2629
8	Beijing	6299	38	standard	2595
9	China	6118	39	news	2522
10	work	6087	40	time	2477
11	put	5422	41	develop	2411
12	wet waste	5284	42	manage	2407
13	community	4995	43	pilot	2384
14	fine	4919	44	advance	2382
15	nationwide	4833	45	finance	2352
16	new	4688	46	street	2339
17	throw	4681	47	ticket	2326
18	Shanghai people	4499	48	enterprise	2314
19	resident	4271	49	drive crazy	2312
20	formal	4252	50	Chengdu	2229
21	recycle	4167	51	takeout	2125
22	environmentally friendly	3937	52	use	2116
23	dry waste	3882	53	publicity	2096
24	Weibo	3837	54	kitchen waste	2062
25	management regulations	3834	55	release	1963
26	eat	3822	56	condition	1962
27	take a video	3565	57	company	1935
28	environment	3143	58	challenge	1931
29	hazardous waste	3108	59	implement	1905
30	citizen	3032	60	society	1900

**Table 2:** High frequency vocabularies, February–March 2020.

No.	Vocabulary	Frequency	No.	Vocabulary	Frequency
1	waste	558	21	Residents′ committees	27
2	classification	402	22	spread	26
3	Shanghai	371	23	dry waste	25
4	community	78	24	tell	25
5	throw	59	25	wet waste	24
6	epidemic	53	26	eat	23
7	life	52	27	take-out	23
8	mask	48	28	someone	23
9	China	45	29	personnel	22
10	really	43	30	law enforcement	21
11	release	42	31	management	21
12	express delivery	40	32	dry	21
13	waste bin	39	33	pour	20
14	Beijing	37	34	national	20
15	isolation	32	35	finally	19
16	scold	31	36	Wuhan	19
17	city	30	37	at home	19
18	residents	30	38	time	19
19	Shanghai people	28	39	virus	18
20	work	28	40	property	18

**Table 3:** High frequency vocabularies in October 2020.

No.	Vocabulary	Frequency	No.	Vocabulary	Frequency
1	waste	161	11	kitchen waste	8
2	classification	113	12	milk tea	7
3	Shanghai	79	13	company	7
4	throw	39	14	Yonghe	7
5	waste can	19	15	purchase	7
6	community	15	16	China	7
7	use	12	17	school	6
8	time	11	18	outside	6
9	Nanjing	11	19	aunt	6
10	common people	8	20	Weibo	6

**Table 4:** Topics and keywords of posts in July 2019.

Topics	Keywords
Topic 1	Waste, classification, Shanghai, life, management regulations, waste bins, throw, community, fines, implementation, city, nationwide
Topic 2	Waste, classification, Shanghai, China, national, implementation, new fashion, pay, pilot country, price, starting from me
Topic 3	Waste, classification, Shanghai, fines, throw wrongly, wet waste, dry waste, implementation, drive crazy, waste bin, fine ticket

**Table 5:** Topics and keywords of posts from August 2019 to December 2020.

Topics	Keywords
Topic 1	Waste, classification, Shanghai, life, management regulations, waste bins, throw, community, fines, implementation, cities, nationwide
Topic 2	Waste, classification, Shanghai, China, national, implementation, new fashion, pay, pilot, power country, price, start from me
Topic 3	Waste, classification, Shanghai, fines, throw wrongly, wet waste, life, dry waste, implementation, drive crazy, waste bin, fine ticket
Topic 4	Waste, classification, wet waste, community, Shanghai, kitchen waste, time, pollution, timed, environment, waste can
Topic 5	Waste, Shanghai, classification, waste can, wet waste, dry waste, eat, aunt, fines, takeaway, community, concert

## Data Availability

The data presented in this study are available on request from the corresponding author.
